# Antimicrobial Activity of Endodontic Medicaments and Vehicles using Agar Well Diffusion Method on Facultative and Obligate Anaerobes

**DOI:** 10.5005/jp-journals-10005-1388

**Published:** 2016-12-05

**Authors:** Triveni M Nalawade, Kishore G Bhat, Suma Sogi

**Affiliations:** 1 PhD Scholar, Department of Pediatric and Preventive Dentistry, KLE Vishwanath Katti Institute of Dental Sciences, Belgaum Karnataka, India; 2 Consutant, Department of Microbiology, KLE University’s Dr. Prabhakar Kore Basic Science Research Centre, Belgaum, Karnataka, India; 3 professor and Head, Department of Pedodontics and Preventive Dentistry, Maharishi Markandeshwar College of Dental Sciences and Research Ambala, Haryana, India

**Keywords:** Agar well diffusion, Medicaments, Minimum inhibitory concentration, Vehicles.

## Abstract

**Aims:**

The aim of this study was to determine the relative antimicrobial effectiveness of these endodontic medicaments and various vehicles using an agar well diffusion assay.

**Materials and methods:**

Double Antibiotic Paste(DAP), modified DAP, 2% Chlorhexidine gluconate and their combination with four vehicles namely Polyethylene glycol 400 (PEG), Propylene glycol (PG), combinations of PG with PEG and lastly Glycerine were tested using agar well diffusion assay. The minimum bactericidal concentration was noted against four standard strains of organisms ie *Streptococcus mutans* ATCC( American Type Culture Collection) 25175, *Staphylococcus aureus* ATCC 12598, *Enterococcus faecalis* ATCC 35550 and *Eschericia coli* ATCC 25922. Successful endodontic therapy depends upon thorough disinfection of root canals. In some refractory cases, routine endodontic therapy is not sufficient, so intracanal medicaments are used for proper disinfection of canals. Issues of resistance, limited spectrum of activity and lack of antifungal properties, the hunt for the ideal intracanal medicament continues. In this regard, the vehicles used to form the pastes play a supportive role by forming the appropriate consistency for placement and may dramatically influence their chemical characteristics like their solubility and diffusion. Thus, inorder to use safer and equally effective intracanal medicaments, Chlorhexidine gluconate is being unveiled in this study.

**Results:**

The difference between the four vehicles when combined with the same endodontic medicament studied above is nonsignificant (NS) except against Porphyromonas gingivalis. Propylene glycol is significantly effective than Glycerine when used with DAP ie C+M medicament combination. (p = 0.029)

**Conclusion:**

2% chlorhexidine gluconate and modified DAP can definitely replace DAP and triple antibiotic paste as end-odontic medicaments with chlorhexidine having an added advantage of bactericidal action, substantivity, biocompatibility, low toxicity, and lesser chances of developing resistance.

**How to cite this article:**

Nalawade TM, Bhat KG, Sogi S. Antimicrobial Activity of Endodontic Medicaments and Vehicles using Agar Well Diffusion Method on Facultative and Obligate Anaerobes. Int J Clin Pediatr Dent 2016;9(4):335-341.

## INTRODUCTION

Dental caries is the most common chronic disease and one of the most expensive diseases to treat. As per the National Oral Health Survey and Fluoride Mapping (2002-2003), there is a very high proportion of untreated caries.^1^ Successful endodontic therapy of the teeth affected with dental caries consists of thorough disinfections of the root canals which cannot be attained by standard treatment alone. Hence, the use of endodontic medicaments for sterilization of root canals especially resistant microbes like *Enterococcus faecalis* has become a necessity. Although calcium hydroxide has been the most used endodontic medicaments, recently triple antibiotic paste (TAP), a combination of ciprofloxacin, metroni-dazole, and minocycline has been proven to be most effective. But, minocycline in TAP has been associated with discoloration and chances of affecting the developing permanent successor if used in deciduous teeth like other tetracyclines cannot be denied.^2^ Appropriate use of the existing antibiotics and also antimicrobial agents and their combinations, which help in decreasing the incidence of resistance development, should be tested and incorporated in the treatment of infectious diseases.

Hence, double antibiotic paste (DAP) being an equally effective alternative, and a combination of amoxicillin clavulanate with metronidazole will be referred to as modified DAP along with 2% chlorhexidine gluconate which will be compared for their antimicrobial effectiveness. To the best of our knowledge, i.e., the first study to compare these three endodontic medicaments, and their combinations with various vehicles are being investigated to find the best combination for various uses in dentistry like intracanal medicaments,^3^ noninstrumentation endodontic therapy (NIET),^4-6^ and local drug delivery as used in periodontal pockets.^7^ The addition of vehicles to these intracanal medicaments not only improves the handling characteristics but also enhances diffusion through den-tinal tubules, antimicrobial activity, and release of the medicaments.^8,9^ The aim of this study was to determine the relative antimicrobial effectiveness of these endodon-tic medicaments and various vehicles using an agar well diffusion assay.

## MATERIALS AND METHODS

This study was carried out in Basic Science Research Centre, Belgaum. It is approved by the Institutional Review Board (Ref no. KLEU/Ethic/14-15/D-73). This study is a part of ongoing *ex vivo* study. The endodontic medicaments evaluated were DAP, modified DAP, 2% chlorhexidine gel and their combination with polyethylene glycol (400 PEG), propylene glycol (PG), combinations of PG with PEG, and glycerine. The antimicrobial activity was carried out against standard strains of American Type Culture Collection (ATCC) against five organisms. *Streptococcus mutans* was chosen as it is the most commonly isolated organism from root canals of infected teeth, whereas *Staphylococcus aureus* and *E. faecalis* were chosen as they are known to develop resistance. *Porphyromonas gingivalis* is also a commonly isolated Gram-negative obligate anaerobe from root canals of teeth and linked to the signs and symptoms of periapical disease and are considered to be more resistant due to outer membrane of their cell-wall structure.^10,11^

### Microorganisms Tested

*Streptococcus mutans* (ATCC 25175)

*Staphylococcus aureus* (ATCC 12598)

*Enterococcus faecalis* (ATCC 35550)

*Porphyromonas gingivalis* (ATCC 33277)

*Escherichia coli* (ATCC 25922).

### Preparations of Microbial Inocula

A direct colony suspension of each test isolate was prepared and the turbidity was adjusted to 0.5 McFarland Standard, for *S. mutans, S. aureus, E. faecalis, E. coli,* and a 1.0 McFarland Standard for *P. gingivalis.*

### Determination of Minimum Inhibitory Concentration of Antimicrobial Substances and their Combinations by Broth Dilution Method

**Figs 1A to D:**
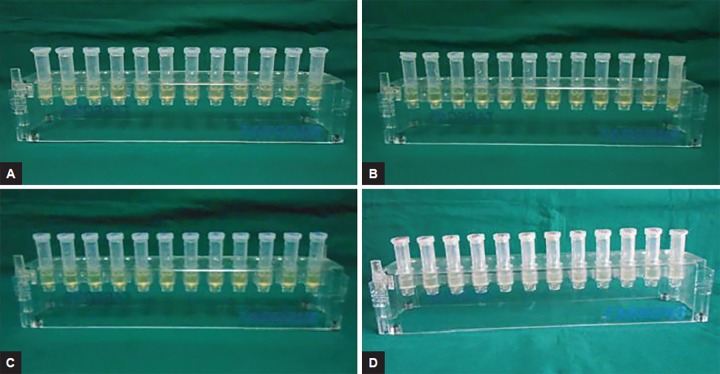
Minimum inhibitory concentration of single antimicrobial substances, i.e.: (A) Chlorhexidine gluconate; (B) ciprofloxacin; (C) amoxicillin clavulanate; and (D) metronidazole by broth dilution method against *P. gingivalis*

**Figs 2A and B:**
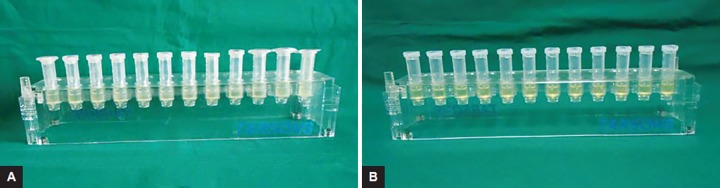
Minimum inhibitory concentration of combination of antimicrobial substances, i.e.: (A) Ciprofloxacin with metronidazole; and (B) amoxicillin clavulanate with metronidazole by broth dilution method against *P. gingivalis*

Minimum inhibitory concentration (MIC) is defined as the lowest concentration where no visible turbidity is observed, i.e., bacteriostatic concentration. Brain heart infusion (BHI) broth was used for the serial dilutions. The selected microorganism was inoculated in BHI broth as per Clinical Laboratory Standard Institute (CLSI) guidelines formerly known as NCCLS, i.e., National Committee for Clinical Laboratory Standards. Also, control strains of *E. coli* ATCC 25922 were kept for monitoring antibacterial susceptibility testing. A known concentrate of the antibacterial substances was serially diluted to two folds in broth and two controls were also included. The first contained undiluted drug which served as the positive control and other contained only inoculums which served as the negative control. Minimum inhibitory concentration was done by broth dilution method first for single drug, namely 2% chlorhexidine gluconate, ciprofloxacin, amoxicillin clavulanate, and metronidazole, and their combinations, i.e., ciprofloxacin with metronidazole (C + M) and amoxicillin clavulanate with metronidazole (A + M). For facultative anaerobes ten serial dilutions were done with incubation time of 24 hours. Whereas for *P. gingivalis,* 12 serial dilutions were done for both single drug and their combinations by broth dilution MIC test with incubation time of 48 hours under anaerobic conditions (Figs 1 and 2). The concentrations used were 0.5 mg/mL of ciprofloxacin, 0.5 mg/mL of amoxicillin clavulanate, and 2 mg/mL of metronidazole for single drug and 1 mg/mL concentration for both the drug combinations, i.e., C + M and A+M.

### Determinations of Antimicrobial Susceptibility using Agar Well Diffusion Method

**Figs 3A to C:**
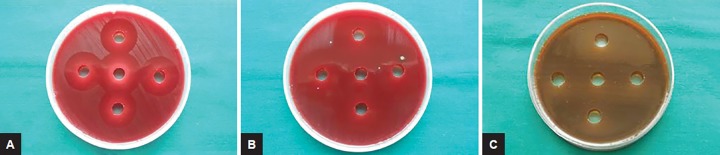
Antimicrobial activity of the endodontic medicaments, i.e.: (A) 2% chlorhexidine gluconate; (B) ciprofloxacin with metronidazole; and (C) amoxicillin clavulanate with metronidazole along with the four vehicle combinations against *P. gingivalis* using agar well diffusion method

Agar well diffusion method was used to determine the antibacterial activity of endodontic medicaments and various vehicles, i.e., DAP, modified DAP, and 2% CHX with PEG, PG, PEG + PG, and glycerine. A 50 μL of respective microbial inoculum was taken using a micropipette in order to provide an even lawn of cells, and loaded onto the agar plates evenly. The agar plates were inoculated with the respective microorganisms by even streaking of the swab over the entire surface of the plate three times rotating the Petri plates at 60° approximately after each applications. Finally, it was swabbed all around the periphery of the agar surface. Five wells of 7 mm size and 4 mm depth were made at an equal distance and 70 μL volume of each medicaments with respective vehicles in the ratio 1:1 (i.e., 35 μL medicaments + 35 μL vehicle) was dispensed into the four wells with the help of micropipettes. The undiluted medicaments, i.e., 70 μL of the antimicrobial substances only were dispensed into the 5th well at the center of inoculated agar plate and were considered as the positive control. The plates were then incubated at 37°C for 24 hours in an aerobic environment for *S. mutans, S. aureus,* and *E. faecalis* and for 48 hours anaerobically for *P. gingivalis* (Figs 3A to C). The Petri plates were observed for zone of inhibition, which were measured using a scale in millimeters. The tests were repeated three times to minimize errors.

### Statistical Analysis

- Mean value and SD
- Kruskal-Wallis test
- Pairwise comparison using *post hoc* Mann-Whitney U test (p < 0.05).

## RESULTS

The least MIC value of all medicaments was that of chlorhexidine and all organisms were resistant to met-ronidazole (Table 1). Combination of ciprofloxacin with metronidazole improved sensitivity of *S. mutans, S. aureus,* and *P. gingivalis* only while amoxicillin clavulanate with metronidazole combination improved susceptibility of *S. mutans* only (Table 2).

All of the selected organisms were susceptible to the antimicrobial drugs and vehicles combination except for *S. mutans,* which exhibited resistance to C + M and all the four vehicle combinations. There was no statistically significant difference in the same antimicrobial drug and vehicles, i.e., PEG, PG, PEG + PG, and glycerine except *P. gingivalis.* There existed significant difference in C + M + PG and C + M + glycerine only on *P. gingivalis.* Considering drug-wise and organism-wise comparison, chlorhexidine was significantly more effective than C + M and A+M against *S. mutans* and *S. aureus.* C + M was the most effective on *E. faecalis* followed by chlorhexidine; whereas A + M was most effective against *P. gingivalis,* thus justifying the combination for obligate anaerobes.

**Table 1:** **Table 1:** Minimum inhibitory concentration of single antimicrobial substances by broth dilution method

*Test agent*		*Microorganism*		*MIC*	
Chlorhexidine		*S. mutans*		0.078%	
		*S. aureus*		0.078%	
		*E. faecalis*		0.156%	
		*P. gingivalis*		0.019%	
		*E. coli*		0.078%	
Ciprofloxacin		*S. mutans*		7.81 μg/mL	
		*S. aureus*		31.25 μg/mL	
		*E. faecalis*		1.95 μg/mL	
		*P. gingivalis*		0.019 μg/mL	
		*E. coli*		1.95 μg/mL	
Amoxicillin clavulanate		*S. mutans*		7.8125 μg/mL	
		*S. aureus*		3.90 μg/mL	
		*E. faecalis*		15.625 μg/mL	
		*P. gingivalis*		0.019 μg/mL	
		*E. coli*		15.625 μg/mL	
Metronidazole		*S. mutans*		500 μg/mL	
		*S. aureus*		500 μg/mL	
		*E. faecalis*		500 μg/mL	
		*P. gingivalis*		0.975 μg/mL	
		*E. coli*		1 μg/mL	

**Table 2:** **Table 2:** Minimum inhibitory concentration of combination of antimicrobial substances by broth dilution method

*Test agent*		*Microorganism*		*MIC*	
Ciprofloxacin + Metronidazole		*S. mutans*		1.95 μg/mL	
		*S. aureus*		7.81 μg/mL	
		*E. faecalis*		1.95 μg/mL	
		*P. gingivalis*		0.039 μg/mL	
		*E. coli*		1.95 μg/mL	
Amoxicillin clavulanate +		*S. mutans*		3.90 μg/mL	
Metronidazole		*S. aureus*		15.625 μg/mL	
		*E. faecalis*		15.625 μg/mL	
		*P. gingivalis*		0.019 μg/mL	
		*E. coli*		15.625 μg/mL	

## DISCUSSION

The sterilization and disinfection of the root canal systems consists of reduction in microbes to enable local response and ensure healing of damaged tissues.^12^ Studies of the microbial diversity of the root canal infections demonstrate that relative proportions of anaerobes increase with time and that facultative anaerobes increase when root canals remain infected for longer periods.^13^ The most predominantly isolated organisms is *S. mutans* followed by facultative microbes, such as *E. faecalis* and *S. aureus,* which constitute the most resistant species.^14^ *E. faecalis* is one of the possible causes of root canal failure.^15^ Recently, *E. faecalis* has been predominantly found in primary end-odontic infections too.^16^ Also, Gram-negative anaerobes, e.g., black-pigmented species have been associated with signs and symptoms of periapical disease and exhibit resistance due to the outer membranes of their cell wall.^15^

The advantage of local applicant of an antibiotic is that it permits the use of very large doses, hence being bactericidal, thus overcoming resistance without risk of systemic toxicity to the subject as the overall dose is small.^13^ Hence, depending on the MIC of combination of drugs by broth dilution method, higher dosage of 100 μg/mL was chosen. Also, previous studies have shown the dose of 100 μg/mL to be bactericidal and no bacteria were recovered from the samples. However, concentrations of 1 and 10 μg/mL allowed some microbes to persist, i.e., these concentrations were only bacteriostatic.^17,18^

Chlorhexidine has been used extensively in endodon-tics for irrigation and as an intracanal medicament and is considered as the gold standard.^19,20^ It is a cationic bigu-anide and an antiseptic. It has substantive properties and even at higher concentrations has very low toxicity.^21,22^ At low concentration, it is bacteriostatic, whereas at high concentration, it is bactericidal.^23^ It shows antimicrobial activity from concentrations as low as 0.1%; and shows bactericidal activity at 2% and is biocompatible.^24,25^

The MIC of chlorhexidine started from 0.019% against *P. gingivalis* to 0.078% against other facultative anaerobes with the highest concentration against *E. faecalis,* i.e., 0.156%. Similar results were observed by Mistry et al,^26^ i.e., < 0.0625% of chlorhexidine against facultative anaerobes but in case of *E. faecalis,* our study shows higher values though the standard strain of *E. faecalis* is the same and the MIC also has been determined in both the studies by broth dilution technique. Strict anaerobes, e.g., *P. gingivalis* as in our study were most susceptible to chlorhexidine without the vehicles, which is contrary to the findings of Filho et al.^20^ Chlorhexidine when studied with vehicles by agar well diffusion method showed lesser zones of inhibition of *P. gingivalis* as compared to other microbes due to being more effective against Gram-positive organisms than Gram-negative.^27^

The MIC of ciprofloxacin alone and decrease in MIC when combination of C + M are in unison with the results stated by Carreira et al^9^ except for *S. aureus* might be due to the use of different ATCC strains. The zones of inhibition of ciprofloxacin against clinical isolates of *S. aureus* and *E. faecalis* are greater at drug concentration of 5 μg/mL^28^ as compared to 100 μg/mL in our study against standard strains of same microorganisms. It is interesting to note that standard strains of *S. mutans* exhibited resistance C + M along with the four vehicles when tested by agar well diffusion method which is in accordance to study by Jain et al.^29^ Double antiseptic paste has been shown to be equally effective as TAP^30^ and it was observed that MIC of C + M against all organisms except *E. faecalis* was lower as compared to ciprofloxacin alone. Another study by Choudhary et al^31^ shows amoxicillin and ciprofloxacin to be highly effective in terms of zones of inhibition, i.e., 31 and 30 respectively, whereas tetracyclines to be moderately effective against clinical isolates of *S. mutans.^31^*

As per the principles of antibiotic therapy, the use of narrow spectra but bactericidal drugs can prevent the development of drug resistance. Combination of two bactericidal drugs can overcome selective pressure and avoid development of resistance in microorganisms. Beta-lactamase production is the most common reason for resistance in Enterobacteriaceae, so clavulanic acid which is a beta-lactamase inhibitor can be used.^32^ Hence, the selection of amoxicillin clavulanate, instead of cipro-floxacin has also been found to be 100% effective against endodontic bacteria.^33^ Therefore, modified DAP with amoxicillin clavulanate was studied for the first time for lesion sterilization and tissue repair or noninstrumenta-tion endodontic technique.

The MIC of amoxicillin clavulanate alone is in accordance with the guidelines by Indian Council Medical Research, except for *E. faecalis* and *E. coli.^32^* Also, the MIC combination of amoxicillin clavulanate with metronidazole improved or stayed consistent for all organisms except *S. aureus.* Additionally, the zones of inhibition of A+M with the four different vehicles were the largest, i.e., against *P. gingivalis* which is similar to findings by Gomes et al.^15^

The zones of inhibition were greater for *P. gingivalis* as obligate anaerobes are rather easily eradicated and met-ronidazole targets anaerobes in particular.^32^ Additionally, 2% CHX was unanimously effective against all pathogens with additional advance of no resistance development being a cation.^34^

**Table 3:** **Table 3:** Antimicrobial activity of the endodontic medicaments and vehicle combinations against selected pathogens using agar well diffusion method

*Antimicrobial drugs*		*Vehicles*		S. *mutans(mm)*		S. *aureus(mm)*		*E. faecalis(mm)*		*P. gingivalis(mm)*	
Chlorhexidine		PEG		31.00 (1.00)		39.00 (1.00)		31.67 (1.52)		26.00 (1.00)	
		PG		32.00 (1.00)		37.67 (1.15)		29.33 (0.57)		27.00 (1.00)	
		PG +PEG		31.33 (0.57)		37.33 (1.52)		29.67 (1.52)		27.33 (0.57)	
		Glycerine		31.33 (0.57)		35.67 (0.57)		28.00 (1.00)		27.67 (1.52)	
		H-Value#		1.96		7.30		6.79		3.37	
		p-value		0.58 (NS)		0.063 (NS)		0.079 (NS)		0.337 (NS)	
C+M		PEG		-		25.00 (1.00)		34.00 (1.00)		37.00 (1.00)	
		PG		-		24.00 (1.00)		33.00 (1.00)		38.33 (0.57) ^a^	
		PG +PEG		-		24.00 (1.00)		33.00 (1.00)		39.00 (1.00)	
		Glycerine		-		23.67 (0.57)		32.67 (0.57)		32.33 (1.52) ^a^	
		H-Value^#^				3.041		3.041		9.00	
		p-value				0.385 (NS)		0.385 (NS)		0.029*	
A+M		PEG		25.00 (1.00)		27.00 (1.00)		26.00 (1.00)		46.00 (1.00)	
		PG		26.33 (0.57)		27.00 (1.00)		25.33 (1.52)		46.33 (1.52)	
		PG +PEG		25.33 (2.08)		26.67 (1.52)		27.33 (.57)		46.00 (1.00)	
		Glycerine		20.00 (1.00)		25.00 (1.00)		28.00 (1.00)		45.00 (1.00)	
		H-Value^#^		7.56		4.44		6.72		2.11	
		p-value		0.056 (NS)		0.217 (NS)		0.081 (NS)		0.548 (NS)	

#Kruskal Wallis test; p > 0.05 non significant (NS); *p < 0.05 significant; ^a^pairwise comparison using post hoc Mann Whitney U test statistically significant p < 0.05). All other pairwise comparisons are non significant (NS)

The use of vehicles like PG not only enhances the penetration of the drugs into their dentinal tubules as observed by Cruz et al,^8^ but also can even make microbes having drug resistance; sensitive when used along with vehicles like PEG, e.g., metronidazole when used with PEG as in study by Carreira et al.^9^ The use of vehicles as carriers for intracanal medicaments also improve handling properties of the resulting paste and enhance their release too. All the above selected vehicles do possess antimicrobial activity.^35^ No significant difference in vehicles when mixed with endodontic medicaments except for PG in comparison with glycerine when used along with C + M combination of drugs against *P. gingivalis* (Table 3) as these vehicles might not be having a synergistic effect when used with the three intracanal medicaments, i.e., 2% chlorhexidine gluconate, C + M and A+M.

The agar well diffusion method or well plate method or the agar diffusion method was used in this study as it is the most commonly used method of antimicrobial activity determination especially of newer substances like plant extracts, new drug formulations, etc. This technique is a well-accepted way of comparing the antibacterial effect of different dental materials, medicaments, etc.^24^ Agar well diffusion was used for the combination of antimicrobial drugs with vehicles as though E-test being the latest method, it is not feasible for combination of drugs.

## CONCLUSION

The many hurdles in using antibiotics as intracanal medicaments are: Issues of resistance, limited spectrum of activity, lack of antifungal properties, and the hunt for the ideal intracanal medicament continues. In this regard, the vehicles used to form the pastes play a supportive role by forming the appropriate consistency for placement and may dramatically influence their chemical characteristics like their solubility and diffusion.^36^ Though difference between the four vehicles studied above is NS except *P. gingivalis,* PG^8^ or PEG^36^ can be used to improve diffusion and slow release of medicaments for longer period of time. The inhibitory effect of PEG is definitely an advantage against Gram-negative species when used as a base for the formulation of endodontic medicaments.^36^

Hence, 2% chlorhexidine gluconate and modified DAP can definitely replace DAP and TAP as endodontic medicaments with chlorhexidine having an added advantage of bactericidal action, substantivity, biocompatibility, low toxicity, and lesser chances of developing resistance.^34,37^ Therefore chlorhexidine can be used for facultatively anaerobic bacterial species, like *S. mutans,* and also for species known to develop resistance like *E. faecalis* and *S. aureus.* Modified DAP was the most effective against obligate anaerobes *P. gingivalis,* but it was also sensitive to 2% chlorhexidine gluconate.

Furthermore, *ex vivo* studies should be carried out as agar diffusion may be influenced by physical-chemical properties of the medicaments, nature of agar, composition, porosity, pH, and thickness of agar media.^38^ *Ex vivo* studies will give us an appropriate understanding of the diffusion through dentinal tubules of these newer combinations of endodontic medicaments and vehicles.
